# Polymorphisms in *SPARC* and Coal Workers' Pneumoconiosis Risk in a Chinese Population

**DOI:** 10.1371/journal.pone.0105226

**Published:** 2014-08-15

**Authors:** Ting Wang, Jingjin Yang, Ruhui Han, Xiaoming Ji, Baiqun Wu, Lei Han, Chen Luo, Jingjing Fan, Baoli Zhu, Chunhui Ni

**Affiliations:** 1 Department of Occupational Medicine and Environmental Health, School of Public Health, Nanjing Medical University, Nanjing, China; 2 Institute of Occupational Disease Prevention, Jiangsu Provincial Center for Disease Prevention and Control, Nanjing, China; Children's National Medical Center, Washington, United States of America

## Abstract

**Background:**

The SPARC is a crucial matricellular protein and may influence the course of various diseases like tumor metastasis and fibrosis. In the present study, we investigated the association between the potential functional polymorphisms in *SPARC* and coal workers' pneumoconiosis (CWP) risk in a Chinese population.

**Methods:**

Five potentially functional polymorphisms (rs1059279, rs1059829, rs1053411, rs2304052 and rs4958281) in *SPARC* were genotyped and analyzed in a case-control study including 697 CWP cases and 694 controls. The genotyping was used by the TaqMan method with the ABI 7900HT Real Time PCR system.

**Results:**

Our results revealed that three SNPs (rs1059279, rs1059829, rs1053411) were significantly associated with increased risk of CWP under an additive model (OR = 1.35, 95%CI = 1.06–1.71, *P* = 0.015 for rs1059279; OR = 1.20, 95%CI = 1.03–1.39, *P* = 0.021 for rs1059829; OR = 1.31, 95%CI = 1.03–1.65, *P* = 0.025 for rs1053411). In the stratification analysis, significant associations were observed between each of these three SNPs and patients with 0–20 pack-years of smoking (OR = 1.73, 95%CI = 1.21–2.45 for rs1059279; OR = 1.48, 95%CI = 1.07–2.05 for rs105982; OR = 1.58, 95%CI = 1.13–2.22 for rs1053411). Furthermore, the association between rs1059279 and CWP risk remained significant among subjects with over 27 years of exposure (OR = 1.27, 95%CI = 1.03–1.56, *P* = 0.023). In the combined analysis of these five polymorphisms, individuals with multiple risk alleles had a higher risk of CWP (*P*trend = 0.015).

**Conclusion:**

Our results indicate that three functional *SPARC* SNPs are associated with an increased risk of CWP in a Chinese population. Further functional research and validation studies with diverse populations are warranted to confirm our findings.

## Introduction

Coal Workers'Pneumoconiosis (CWP) is a serious occupational disease derived from inhalation and deposition of occupational coal mine dust and/or silica particulates in the lungs. The exposure may lead to the aberrant proliferation of activated fibroblasts, pathological remodeling, and excessive deposition of extracellular matrix (ECM) in lung tissues [1], [2]. Currently, there is no effective treatment of CWP. In China, 87.72% of the reported occupational cases were attributed to pneumoconiosis in 2013, of which CWP (60.28%) and silicosis (34.96%) accounted for the majority [3]. The incidence and progression of CWP are related to both dust exposure levels and silica content in the dust [4]. However, only a portion of individuals exposed to coal dust or silica develop CWP in their lifetime, suggesting that genetic susceptibility factors also play a role in the development of CWP [5].

SPARC (secreted protein, acidic and rich in cysteine), also known as osteonectin or BM-40, is a 43 kilo-Dalton matricellular glycoprotein which is secreted into the extracellular space along with other extracellular matrix components, including collagens, but does not serve a structural function in the ECM [6]. SPARC participates to not only regulate cell-cell and cell-matrix interactions, but also modify ECM deposition, influence angiogenesis, and alter the activity of a number of cytokines and growth factors [7], such as stimulating the transforming growth factor beta (TGF-β) signaling system [8]. In addition, SPARC-null mice display a lesser amount of pulmonary fibrosis compared with wild type mice in animal models of bleomycin-induced pulmonary fibrosis [9]. These points indicate that SPARC may play an important role in tissue fibrosis.

Single nucleotide polymorphisms (SNPs) are the most frequent sequence variations in the human genome. The SPARC gene is located on human chromosome 5q31-32 [10], and there are at least 800 SNPs of SPARC reported in the SNP database (http://www.ncbi.nlm.nih.gov/snp). Most of these are located in introns. SNPs located in exon regions may alter protein function, whereas SNPs in the gene promoter modify gene expression at transcription levels. SNPs in the 3′-untranslated region (3′-UTR) may be regulated by microRNA at translational levels.

There are a total of ten SNPs in the functional regions of the SPARC: one (rs2304052) in the exon, one (rs4958281) in 5′-UTR, and eight in 3′-URT with a minor allele frequency (MAF) greater than 0.05 in the Chinese population. Therefore, we selected the two SNPs located in the exon and 5′-UTR. Among the eight SNPs in 3′-UTR, three SNPs (rs1059279, rs1059829 and rs1053411) were selected based on a linkage coefficient of r^2^>0.80. To date, there is no literature on genetic variants in SPARC and their roles in susceptibility to CWP. Therefore, the current study aimed to investigate the associations of five functional SNPs in SPARC and the susceptibility to CWP in the Chinese population, as well as their impact on the progression of the disease.

## Materials and Methods

### Study subjects

From January 2006 to December 2010, 694 healthy males and 697 males with definite CWP were recruited from the coal mines of Xuzhou Mining Business Group Co., Ltd., as described previously [11]. All subjects in our study were ethnic Han Chinese without a direct family/genetic relationship. All studied individuals were selected from the same mines. Subjects were excluded if they had clinical evidence of autoimmune diseases, had received immunosuppressive or immunostimulatory therapy, or were subjected to radiotherapy. High kilovolt chest X-rays and physical examinations were performed to confirm diagnoses based on the China National Diagnostic Criteria for Pneumoconiosis (GBZ 70-2002). These criteria are identical to the 1980 International Labour Organization (ILO) in the judgment of opacity profusion [4], [12]. Two independent physicians (Z. Song and X. Jia) assessed the chest X-rays. Pneumoconiosis cases were classified into stage I, stage II or stage III according to the size, profusion, and opacity distribution range. Controls were recruited from healthy subjects who were seeking outpatient physical examinations at the hospital department, and were frequency matched on age (±5 years), dust exposure period, and occupation. Using a double-blind investigation method, live interviewers conducted participant questionnaires. The epidemiological questionnaire focused on age, respiratory symptoms, occupational histories, and smoking habits. Five milliliter blood samples were obtained from all subjects and used for routine laboratory tests. A written informed consent was obtained from each subject before participating in the study. The protocol and consent form were approved by the Institutional Review Board of Nanjing Medical University (FWA00001501).

### SNPs selection

To select the most likely functional SNPs influencing the *SPARC* gene, we selected the SNPs located in the exon, 3′-UTR, and 5′-UTR, as determined in the HapMap Genome Browser release (Phase 1 & 2 release - full dataset). We included the following criteria for SNPs: (i) the SNPs should be located in the exon, 3′UTR and 5′UTR, (ii) the minor allele frequency (MAF) should be >5% in the Chinese Han Beijing population (CHB), and (iii) in the case of multiple SNPs in the same haplotype block (linkage coefficient r^2^>0.8 in CHB of 1000 Genome database), only the most representative SNP was selected. Ultimately, three SNPs (rs1059279, rs1053411, and rs1059829) located in 3′UTR, one SNP rs2304052 in the exon, and one SNP rs958281 located in 5′UTR were included in the study. The LD results of these five chosen SNPs demostrated that rs1059829 had a medium linkage disequilibrium with rs1053411 (RSquared = 0.786), however, the rs1059279 was not included in 1000 Genome database.

### Genotyping

Genomic DNA was extracted from leukocyte pellet by proteinase K digestion and followed by phenol-chloroform extraction and ethanol precipitation. Genotyping was performed using the TaqMan method with the ABI 7900 real-time PCR system (Applied Biosystems, Foster City, CA, USA). SDS allelic discrimination software (version 2.4, provided by ABI) was used for analysis of genotyping results.

The sequences of the primers and probes for each SNP are available on request. Further detailed information about the sequence of each primer and probe is listed in Table S1. Amplification was performed in a total volume of 5 µl, containing 50 ng of genomic DNA, 2.5 µl of Mix, 0.25 µl of each primer, 0.125 µl of each probe and 1.25 µl Nuclease-Free Water, under the following conditions: 50°C for 2 minutes and 95°C for 10 minutes followed by 45 cycles of 95°C for 15 seconds and 60°C for 1 minute. Negative controls were included in each plate to ensure accuracy of genotyping. For quality control, genotyping was done in a blinded fashion without knowledge of the workers' personal details or case/control status of subjects. Furthermore, a random 10% of cases and controls were genotyped twice by different individuals, with a reproducibility of 100%. All relevant data are within Data S1.

### Statistical analyses

Deviations of the characteristics for CWP patients and control subjects were examined by the Student-*t* test (for continuous variables) or the *χ^2^* test (for categorical variables). Differences in allele and genotype frequencies of the two groups were assessed using the Pearson χ2 test or Fisher's exact test. Unconditional multivariate logistic regression analyses adjusting for age, exposure years, pack-years smoked, and occupation, were used to estimate ORs and the 95% confidence interval (CI) for assessing the strength of association between the polymorphisms in *SPARC* and risk of coal workers' pneumoconiosis under various genetic models. These were defined as Aa versus AA and aa versus AA for co-dominant, aa+Aa versus AA for dominant, aa versus AA+Aa for recessive, and a versus A for additive model (A: major allele, a: minor allele). Hardy-Weinberg equilibrium (HWE) was tested using a goodness-of-fit χ^2^-test. For the stratified analysis, the dust-exposure cut-off used was based on median dust-exposure years of the recruited patients and controls. Genotypes were coded as wild types (major-allele homozygote) and variants (minor-allele homozygote and heterozygote). *P*-values of less than 0.05 were considered statistically significant. All statistical tests were two-sided and were analyzed with R software (version 3.0).

## Results

Demographic and clinical information is summarized in Table 1. No significant differences were observed for age (*P* = 0.103), exposure years (*P* = 0.105), or occupation (*P* = 0.534) between CWP patients and controls. The distribution of smoking status between cases and controls was parallel (*P* = 0.250). As expected, smoking amount (pack- years) in CWP cases was significantly more than that of controls (*P*<0.001). Pneumoconiosis stages from I to III were identified as 59.5%, 31.4%, and 9.0%, respectively.

**Table 1:** Demographic and selected variables among the CWP cases and control subjects.

Variables	CWP(n = 697)		Controls(n = 694)		*P*
	N	%	N	%	
Age, years (mean±SD)	68.0±11.1		67.1±8.4		0.103
Exposure years (mean±SD)	26.6±9.0		27.3±7.8		0.105
Smoking status					
Never	340	48.8	360	51.9	0.250
Ever	357	51.2	334	48.1	
Former	163	23.4	91	13.1	
Current	194	27.8	243	35.0	
Pack-years smoked					<0.001
0	340	49.2	360	52.6	
0–20	223	32.0	132	19.0	
>20	134	19.2	202	29.1	
Occupation					0.534
Tunnel and coal mining	663	95.1	652	94.0	
Transport	16	2.3	17	2.5	
Others	18	2.6	25	3.6	
Stage					
I	415	59.5			
II	219	31.4			
III	63	9.0			

Details for the SNPs detected in our study are summarized in Table 2. All genotyped distributions of control subjects were consistent with those expected from the HWE. The MAFs of these five polymorphisms were consistent with those reported in the HapMap database.

**Table 2:** Primary information of genotyped SNPs.

Cluster ID	Region	dbSNP allele	MAF			HWE^b^
			dbSNP^a^	Case	Control	
rs1059279	3′UTR	A>C	0.33	0.33	0.30	0.410
rs4958281	5′UTR	C>T	0.08	0.04	0.05	0.693
rs2304052	Exon	T>C	0.07	0.07	0.08	0.297
rs1059829	3′UTR	C>T	0.48	0.39	0.35	1
rs1053411	3′UTR	C>G	0.33	0.34	0.30	0.929

dbSNP^a^ based on Asian in dbSNP;

HWE^b^ (Hardy–Weinberg equilibrium) *P* value of the control group.

Furthermore, we performed a multivariate logistic regression analysis to assess the effect of each SNP on CWP risk (adjusting for age, exposure years, pack-years smoked, and occupation). Parameters for the association of SNPs with CWP are shown in Table 3. There are three SNPs (rs1059279, rs1059829, rs1053411) significantly associated with the risk of CWP. Analysis under different genetic models revealed that the risk allele increased the susceptibility to CWP under co-dominant (OR = 1.63, 95%CI = 1.12–2.37, *P* = 0.011 for CC versus AA for rs1059279; OR = 1.56, 95%CI = 1.13–2.17, *P* = 0.007 for TT versus CC for rs1059829; OR = 1.49, 95%CI = 1.04–2.14, *P* = 0.029 for GG versus CC for rs1053411), recessive (OR = 1.53, 95%CI = 1.07–2.19, *P* = 0.021 for rs1059279; OR = 1.52, 95%CI = 1.12–2.06, *P* = 0.007 for rs1059829; OR = 1.43, 95%CI = 1.01–2.01, *P* = 0.041 for rs1053411), and additive (OR = 1.2, 95%CI = 1.02–1.41, *P* = 0.027 for rs1059279; OR = 1.20, 95%CI = 1.03–1.39, *P* = 0.021 for rs1059829; OR = 1.17, 95%CI = 1.00–1.38, *P* = 0.044 for rs1053411) models. These associations remained significant after adjusting for age, exposure years, pack-years smoked, and occupation, except for the correlation between rs1059829 and CWP risk under an additive model. As for rs2304052 and rs4958281, none of the genetic models showed any effect of the minor allele on the development of CWP.

**Table 3:** Distributions of genotypes and their associations with risk of CWP.

Variables	CWP cases		Controls		OR (95%CI)^a^		*P* a	OR (95%CI)^b^		*P* b
	N	%	N	%						
rs1059279	n = 692		n = 689							
AA	309	44.8	337	48.9	1.00			1.00		
AC	301	43.5	297	43.1	1.11	0.88–1.38	0.378	1.11	0.89–1.39	0.340
CC	82	11.7	55	8.0	1.63	1.12–2.37	0.011	1.63	1.12–2.38	0.011
Dominant					1.18	0.95–1.46	0.126	1.25	0.91–1.71	0.163
Recessive					1.53	1.07–2.19	0.021	2.41	1.34–4.33	0.003
Additive					1.2	1.02–1.41	0.027	1.35	1.06–1.71	0.015
rs4958281	n = 692		n = 689							
CC	633	91.4	621	90.1	1.00			1.00		
TC	59	8.5	66	9.6	0.88	0.61–1.27	0.485	0.88	0.60–1.27	0.481
TT	0	0	2	0.3	----	----	----	----	----	----
Dominant					0.85	0.59–1.23	0.388	0.63	0.37–1.06	0.083
Recessive					0.00	0.00-inf	0.999	0.00	0-inf	0.999
Additive					0.83	0.58–1.19	0.306	0.621	0.37–1.04	0.070
rs2304052	n = 692		n = 689							
TT	593	85.7	587	85.2	1.00			1.00		
CT	95	13.7	96	13.9	1.00	0.72–1.33	0.895	0.98	0.72–1.34	0.909
CC	4	0.6	6	0.9	0.66	0.19–2.35	0.521	0.60	0.17–2.16	0.438
Dominant					0.96	0.71–1.30	0.793	0.86	0.55–1.34	0.515
Recessive					0.66	0.19–2.36	0.524	0.48	0.08–2.96	0.426
Additive					0.95	0.72–1.25	0.699	0.85	0.56–1.28	0.436
rs1059829	n = 690		n = 687							
CC	270	39.1	293	42.6	1.00			1.00		
TC	302	43.8	312	45.4	1.05	0.84–1.32	0.674	1.05	0.84–1.13	0.650
TT	118	17.1	82	11.9	1.56	1.13–2.17	0.007	1.56	1.13–2.17	0.008
Dominant					1.16	0.93–1.43	0.184	1.18	0.86–1.62	0.319
Recessive					1.52	1.12–2.06	0.007	1.72	1.09–2.72	0.021
Additive					1.20	1.03–1.39	0.021	1.24	0.99–1.55	0.059
rs1053411	n = 692		n = 689							
CC	308	44.5	333	48.3	1.00			1.00		
GC	297	42.9	293	42.5	1.10	0.88–1.37	0.422	1.09	0.88–1.37	0.423
GG	87	12.6	63	9.1	1.49	1.04–2.14	0.029	1.48	1.03–2.13	0.032
Dominant					1.17	0.94–1.44	0.155	1.25	0.91–1.71	0.165
Recessive					1.43	1.01–2.01	0.041	1.99	1.17–3.39	0.011
Additive					1.17	1.00–1.38	0.044	1.31	1.03–1.65	0.025

a Two-sided x^2^ test.

b Adjusted for age, exposure years, pack-years smoked, and occupation.

The associations between each of these three SNPs (rs1059279, rs1059829, rs1053411) and CWP risk were further stratified by exposure years and pack-years smoked. As shown in Table 4, the association between rs1059279 and CWP risk remained significant among subjects who had greater than 27 years of exposure (OR = 1.27, 95%CI = 1.03–1.56, *P* = 0.023) and individuals with 0–20 pack-years smoked (OR = 1.73, 95%CI = 1.21–2.45, *P* = 0.002) under an additive model. In addition, the variants rs1059829 and rs1053411 both also significantly increased CWP risk of individuals with 0–20 pack-years smoked (OR = 1.48, 95%CI = 1.07–2.05, *P* = 0.019 for rs1059829; OR = 1.58, 95%CI = 1.13–2.22, *P* = 0.008 for rs1053411) under an additive model. The function prediction results of these three SNPs are listed in Table 5.

**Table 4:** Stratification analyses between the genotypes of three SNPs and CWP risk.

Variables	rs1059279				rs1059829				rs1053411			
	cases^a^	controls^a^	OR (95%CI)^b^	P^b^	cases^a^	controls^a^	OR (95%CI)^b^	P^b^	cases^a^	controls^a^	OR (95%CI)^b^	P^b^
Exposure years												
<27	28/110/132	16/120/129	1.11(0.85–1.45)	0.447	43/114/112	29/119/117	1.17(0.91–1.50)	0.210	31/109/130	21/115/129	1.10(0.85–1.43)	0.470
≥27	54/191/177	39/177/208	1.27(1.03–1.56)	0.023	75/188/158	53/193/176	1.21(1.00–1.47)	0.052	56/188/178	42/178/204	1.21(1.00–1.49)	0.059
Pack–years smoked												
0	40/135/165	31/156/171	1.05(0.84–1.32)	0.664	62/136/141	46/163/148	1.11(0.90–1.37)	0.331	41/140/159	36/152/170	1.05(0.84–1.32)	0.638
0–20	27/103/89	7/51/73	1.73(1.21–2.45)	0.002	37/103/79	12/57/61	1.48(1.07–2.05)	0.019	31/97/91	8/53/70	1.58(1.13–2.22)	0.008
>20	15/63/55	17/90/93	1.20(0.85–1.69)	0.293	19/63/50	24/92/84	1.15(0.83–1.59)	0.414	15/60/58	19/88/93	1.10(0.79–1.54)	0.572

a Variant homozygote/Heterozygote/Wild type homozygote;

b Adjusted for age, exposure years, pack-years smoked, and occupation.

**Table 5:** Function prediction of SNPs.

	regolumeDB	eQTL	Dnase	TFBS	H3K27ac	miRNA-LOSS
rs1059829	1d	Y	Y	Y	Y	hsa-miR-541-5p
rs1059279	3a	N	Y	Y	Y	
rs1053411	4	N	N	N	Y	hsa-miR-4311

To deeply evaluate potential interactions of *SPARC* gene polymorphisms on the risk of CWP, we combined five polymorphisms based on the number of variant (risk) alleles (i.e.: rs1059279C, rs4958281T, rs2304052C, rsrs1059829T, rsrs1053411G). As shown in Table 6, individuals with multiple risk alleles did have a higher risk of CWP (*P*trend = 0.015).

**Table 6:** Frequency distributions of the combined genotypes between CWP cases and controls.

	CWP cases		Controls		OR (95%CI)^a^	P	OR (95%CI)^b^	P
Risk Allele	N	%	N	%				
2∼4	271	39.28	288	41.92	1		1	
5∼7	299	43.33	323	47.02	0.98(0.78–1.24)	0.888	0.99(0.79–1.25)	0.937
8∼10	120	17.39	76	11.06	1.68(1.20–2.34)	0.002	1.68(1.20–2.34)	0.002
P_trend						0.016		0.015

a Variant homozygote/Heterozygote/Wild type homozygote;

b Adjusted for age, exposure years, pack-years smoked, and occupation.

## Discussion

SPARC, a matricellular protein secreted from several different cell types into the ECM, may modulate interactions between cells and the surrounding ECM [6]. The elevated expression of SPARC has been reported in animal models of fibrotic disease and in human fibrotic tissues, including heart [13], lungs [14], kidneys [15] and others. However, in patients with pulmonary fibrosis, the expression of SPARC was found to be increased and was localized to the cytoplasm of pulmonary fibroblasts [16]. SPARC participates in collagen deposition probably through three mechanisms in the pathogenesis of pneumoconiosis. First, SPARC might bind procollagen and prevent its interaction with cellular receptors, such as the discoidin domain receptor (DDR) 2 and integrin α2β1 [6]. In the absence of SPARC, procollagen accumulates at the cell surface, resulting in less total collagen and fewer thick collagen fibers. Second, there is a reciprocal regulatory mechanism between SPARC and TGF-β. Blocking of TGF-β signaling by the ALK-5 inhibitor SB-525334 significantly decreases SPARC expression as well as the degree of fibrosis in vivo [17], whereas decreased SPARC expression leads to decreased TGF-β activity [18]. Last, SPARC may activate nuclear localization of β-catenin and integrin-linked kinase (ILK) [19]. The activation of β-catenin in fibroblasts promotes stabilization of the myofibroblast phenotype and an anti-apoptotic phenotype, while the activation of ILK leads to ROS production, one of the causative factors of recurrent epithelial damage in fibrotic lungs [14], [20].

Several mouse models have confirmed that SPARC is affiliated with pulmonary fibrosis. Savani et al. [21] used bleomycin sulfate infused intra-tracheally at 0.15 U/mouse to cause a fibrotic response in WT and SPARC-null mice. The outcome revealed that SPARC-null mice had increased tissue destruction and increased inflammatory cell recruitment, specifically neutrophils, in comparison to bleomycin-treated WT mice. These findings were consistent with Sangaletti's study [22]. The reasons behind the outcome are not readily apparent, but SPARC could be produced by both bone marrow-derived and lung fibroblasts, and different sources might play a different role [23]. Sangaletti used bone marrow chimeric mice and found that expression of SPARC in pulmonary fibroblasts promoted collagen deposition, while the expression of SPARC in bone marrow cells impeded inflammatory infiltrates. This elaborate study demonstrated the intricate association between fibrosis and inflammation [23].

It is well known that genetic and environmental factors are involved in the development of CWP. To our knowledge, this is the first evaluation of the association between functional SNPs in *SPARC* and pneumoconiosis susceptibility in a Chinese population. Statistical analyses identified three SNPs (rs1059279, rs1059829, rs1053411) that were significantly associated with pneumoconiosis. In addition, the rs1059279 was not included in 1000 Genome database when we search for LD in SNP selection process. However, we found rs1059279 was in high linkage disequilibrium with rs1053411 (RSquared = 0.904) using our own genotyped data. Furthermore, stratification analyses were applied and hinted that each of these three SNPs (rs1059279, rs1059829, and rs1053411) significantly increased CWP risk of individuals with 0-20 pack-years smoking.

Furthermore, we speculate on the function of three SNPs: rs1053411 might affect the miRNA-LOSS of hsa-miR-4311, while rs1059829 might affect the miRNA-LOSS of hsa-miR-541-5p, which could well be involved in the regulation of mRNA production and stability. Moreover, rs1059829 was a locus of expression for Quantitative Trait Loci (eQTL) and Transcription Factor Binding Site (TFBS), thus could affect transcription activity and even consequently predispose individuals to excessive fibrogenesis. The relevant TFBS include Nfκb1, Interferon regulatory factor 4 (IRF4), B-cell CLL/lymphoma 3 (BCL3), and so on, all of which have innumerable links to the molecular mechanisms that result in the transcriptional activation of genes responsible for the fibrotic process. These findings set new insights into the role of SPARC in the pathogenesis of pneumoconiosis.

Several limitations of this study should be addressed. First, the possibility of selection bias of subjects could not be ruled out in this population-based, case-control study. Second, our sample size was only moderate, further studies are required to replicate our results in larger and more diverse ethnic populations. Third, since five SNPs were tested, one might apply an appropriate multiple testing correction, such as the Bonferroni correction, otherwise the significant association between these three SNPs and CWP risk should be interpreted with caution.

In conclusion, the present study indicates that three functional *SPARC* SNPs are associated with an increased risk of CWP in a Chinese population. Further functional research and validation studies with diverse populations are warranted to confirm our findings.

## Supporting Information

Table S1 **The sequences of the primers and probes for each SNP.**
(DOC)

Data S1 (XLS)
