# Supplementary material for: Polymorphisms in SPARC and Coal Workers' Pneumoconiosis Risk in a Chinese Population
Source: PLoS One. 2014 Aug 15;9(8):e105226. doi: 10.1371/journal.pone.0105226 (PMC4134282; doi:10.1371/journal.pone.0105226)
Supplement: Table S1 — The sequences of the primers and probes for each SNP. (DOC) [file pone.0105226.s001.doc]

Supplementary Table1. The sequences of the primers and probes for each SNP

| SNP_ID | Primers for PCR amplification (5’–3’) | Probes for PCR amplification (5’–3’) |
| --- | --- | --- |
| rs1059279 | F:CCGACCATCCCATTAACTTTGA  R:TGTCATAAGGTTTTTAGCATGTTCCT | FAM:ATTAATAGAAGAAAAAAGG-MGB  HEX:ATTAATAGAAGAACAAAGGG-MGB |
| rs4958281 | F:AGCTCTGGGCTGGGTCTATCT  R:AAATGAAAGACAGAGACAGCTTTGG | FAM:TCCTTCCCCCGGCC-MGB  HEX:CTCCTTCCCCTGGC-MGB |
| rs2304052 | F:TTCCACCACCTCTGTCTCATCA  R:TCTGAACGCTACTTTCTCTTTCCA | FAM:CAGGGCTTCTTGC-MGB  HEX: AGGGCCTCTTGCT-MGB |
| rs1059829 | F:CCAGGAAGGCAGTTTCTAAGTCATT  R:TTAATCATACAAACTCTCACCATGATGT | FAM:TTTTATTTTGAAAGATTTGTG-MGB  HEX: TTTATTTTGAAGGATTTGTG-MGB |
| rs1053411 | F:CAGAACAACAAACCATCCAAACATT  R:TTGTGATCTAAATCCACTCCTTCCA | FAM: TTAAAGACAGAATCCG-MGB  HEX: TTAAAGAGAGAATCCG-MGB |

They were designed and manufactured by BioSteed BioTechnologies Co.,Ltd. (Nanjing, China).
